# Living with trimethylaminuria and body and breath malodour: personal perspectives

**DOI:** 10.1186/s12889-024-17685-w

**Published:** 2024-01-18

**Authors:** Cole C. Flaherty, Ian R. Phillips, Azara Janmohamed, Elizabeth A. Shephard

**Affiliations:** 1 Warwick Medical School, University of Warwick, Coventry, UK; 2 Department of Structural and Molecular Biology, University College London, London, UK; 3 School of Biological and Behavioural Sciences, Queen Mary University of London, London, UK; 4 Department of Clinical Pharmacology, St. Mary’s Hospital, Imperial College Healthcare NHS Trust, London, UK

**Keywords:** Body and breath malodours, Discrimination, Loneliness, Social isolation, Survey, Trimethylaminuria

## Abstract

**Background:**

Many people suffer from body and breath malodour syndromes. One of these is trimethylaminuria, a condition characterized by excretion in breath and bodily fluids of trimethylamine, a volatile and odorous chemical that has the smell of rotting fish. Trimethylaminuria can be primary, due to mutations in the gene encoding flavin-containing monooxygenase 3, or secondary, due to various causes. To gain a better understanding of problems faced by United Kingdom residents affected by body and breath malodour conditions, we conducted a survey.

**Methods:**

Two anonymous online surveys, one for adults and one for parents/guardians of affected children, were conducted using the Opinio platform. Participants were invited via a trimethylaminuria advisory website. Questions were a mix of dropdown, checkbox and open-ended responses. Forty-four adults and three parents/guardians participated. The dropdown and checkbox responses were analysed using the Opinio platform.

**Results:**

All participants reported symptoms of body/breath odour. However, not all answered every question. Twenty-three respondents experienced difficulties in being offered a diagnostic test for trimethylaminuria. Problems encountered included lack of awareness of the disorder by medical professionals and reluctance to recognise symptoms. Of those tested, 52% were diagnosed with trimethylaminuria. The main problems associated with living with body/breath malodours were bullying, harassment and ostracism in either the workplace (90%) or in social settings (88%). All respondents thought their condition had disadvantaged them in their daily lives. Open-ended responses included loss of confidence, stress, exclusion, isolation, loneliness, depression and suicidal thoughts. Respondents thought their lives could be improved by greater awareness and understanding of malodour conditions by medical professionals, employers and the general public, and appreciation that the malodour was due to a medical condition and not their fault.

**Conclusions:**

Breath and body malodour conditions can cause immense hardship and distress, both mentally and socially, having devastating effects on quality of life. It would be advantageous to establish a standardised pathway from primary care to a specialist unit with access to a robust and reliable test and diagnostic criteria. There is a need to recognise malodour disorders as a disability, giving affected individuals the same rights as those with currently recognised disabilities.

## Background

Many people are affected by body malodour syndromes, one of which is trimethylaminuria (TMAU), a condition that manifests itself in body and breath malodours [1, 2]. Both men and women are affected by TMAU. Women, however, report increased and intense body odour during menstruation, which exacerbates the condition [3]. The odour is due to trimethylamine (TMA), which is produced by commensal gut bacteria from ubiquitous dietary components such as choline and carnitine [4, 5]. TMA is the chemical that gives rotting fish its unpleasant smell and the human olfactory system has evolved to recognise this smell with high acuity, detecting it at concentrations as low as 1 part per billion. There are two forms of the disorder: primary TMAU and secondary TMAU [6, 7]. Clinical presentations of these forms can overlap.

Primary TMAU is of genetic origin and can be considered as an inborn error of metabolism. It is a consequence of homozygous or compound heterozygous mutations in the *FMO3* gene [8–10], which encodes the protein flavin-containing monooxygenase 3 (FMO3). In non-affected people, the enzyme FMO3 converts odorous TMA to the non-odorous trimethylamine *N*-oxide (TMAO) in the liver. TMAO travels in the systemic circulation and is excreted in urine [9, 11]. Affected individuals have a defect in their ability to convert TMA to TMAO. The unmetabolized TMA is excreted in the urine, sweat and other bodily fluids, and this is what causes the body and breath odour problems [1, 2]. Several genetic mutations in the *FMO3* gene have been identified that affect the ability of the FMO3 enzyme to convert TMA to TMAO [12–14] and are causative of primary TMAU. The severity of the disorder is related to the extent to which a mutation affects the function of FMO3 [15]. The incidence of heterozygotes (carriers) in the white British population is 0.5 to 1.0% [16]. The pattern of inheritance is autosomal recessive, giving an estimated incidence of affected individuals as high as 1 in 40,000 [17].

Secondary TMAU is more common than primary TMAU [15]. Although there is no single cause of secondary TMAU, individuals are thought to have an imbalance of the gut microbiome, known as dysbiosis [6]. This results in the over-production of TMA in the gut, which overwhelms the oxygenating capacity of FMO3. Subsequently, this results in accumulation of TMA and its secretion causes body odour symptoms. Some individuals acquire a body odour disorder following certain illnesses and infections that affect the ability of FMO3 to convert TMA to TMAO [1, 6].

Clinically, TMAU is diagnosed by measuring urinary excretion of TMA as the percent of total TMA (i.e., TMA plus TMAO) excreted as unmetabolized free TMA (TMA/TMA + TMAO) [6, 18]. If more than 40% of total TMA is excreted as unmetabolized free TMA the condition is categorized as severe TMAU. If 10–39% of total TMA is excreted as unmetabolized free TMA the condition is categorized as mild TMAU. Unaffected individuals excrete 0–9% of total TMA as unmetabolized free TMA [7].

There are several reports, both anecdotal and published, based on single or small numbers of individuals, on the difficulties faced in living with TMAU [1, 19–21]. During bouts of body odour those who suffer from primary or secondary TMAU can be victimised and discriminated against. Although the disorder is not life-threatening, suicidal tendencies have been reported [1]. To gain a better understanding of the problems faced by United Kingdom (UK) individuals who present with body odour, we have conducted a survey, in collaboration with the patient advisory group Metabolic Body Odours (MEBO). We explored the influence that body and breath malodour has on daily life, both in seeking medical help and in the settings of education, employment and social activity. The survey provides both qualitative and quantitative information and reveals that affected individuals experience frustration, discrimination and disregard, which can lead to feelings of isolation and loneliness.

## Methods

The aim of the study was to identify problems faced by individuals affected by TMAU or other body/breath malodours. The project was approved by the University College London (UCL) Research Ethics Committee, Approval ID Number: 14,227/001. Two surveys were designed, one for adults and one for parents or guardians of affected children. The anonymous surveys were designed using the web-based Opinio platform (Opinio 7, ObjectPlanet, Inc.) hosted by UCL. The adult survey comprised 22 questions and the parent/guardian survey 19 questions. Questions were a mix of dropdown, checkbox and open-ended. A focus group, assembled by the patient advisory group MEBO, checked and approved the questions. An invitation to participate in the surveys entitled ‘Living with Trimethylaminuria’ was posted on a temporary MEBO website, together with a participant information sheet, which included the names and contact details of those carrying out the research, an invitation paragraph, the purpose of the project and confirmation that the questionnaire was anonymous. To qualify, participants were asked to confirm that they were aged 18 or older and were resident in the UK. The survey remained open for 6 weeks. The dropdown and checkbox responses were analysed using the Opinio platform.

## Results

### Demographics of respondents

Forty-four adults, aged 18 or older, participated in the questionnaire. All confirmed they were a UK resident: 36 were resident in England, 6 in Scotland, 1 in Wales and 1 in Northern Ireland. Forty-three specified a gender, 34 female and 9 male, and one chose the response ‘prefer not to say’ (Table 1).

### Age of onset of TMAU or body/breath odour

All individuals reported symptoms of body/breath odour. The onset of the symptoms ranged from 5 to 60 years of age, with the median being 19 (Table 1).

**Table 1:** Age symptoms presented, gender and diagnosis

Age symptoms presented (year)	Gender selected	Diagnosis
5	Male	Yes, primary
14	Female	Yes, primary
29	Female	Yes, primary
32	Female	Yes, primary
60	Female	Yes, primary
8	Female	Yes, secondary
14	Male	Yes, secondary
16	Female	Yes, secondary
17	Female	Yes, secondary
17	Male	Yes, secondary
23	Female	Yes, secondary
35	Female	Yes, secondary
35	Female	Yes, secondary
40	Female	Yes, secondary
42	Female	Yes, secondary
16	Female	TMAU1/2?
6	Male	No diagnosis
6	Male	No diagnosis
11	Female	No diagnosis
11	Female	No diagnosis
11	Prefer not to say	No diagnosis
12	Female	No diagnosis
13	Female	No diagnosis
14	Female	No diagnosis
14	Male	No diagnosis
15	Female	No diagnosis
15	Female	No diagnosis
17	Female	No diagnosis
18	Female	No diagnosis
18	Female	No diagnosis
20	Female	No diagnosis
21	Male	No diagnosis
23	Female	No diagnosis
23	Female	No diagnosis
24	Female	No diagnosis
26	Female	No diagnosis
26	Female	No diagnosis
26	Male	No diagnosis
28	Female	No diagnosis
30	Female	No diagnosis
30	Female	No diagnosis
34	Female	No diagnosis
37	Male	No diagnosis
39	Female	No diagnosis

### TMAU diagnosis

TMAU can be diagnosed by urine and genetic tests. Urine tests measure urinary concentrations of TMA and TMAO [6, 18]. A genetic test screens for mutations in the *FMO3* gene [7]. Thirty-two of the respondents (73%) had a urinary test for TMAU. Of these, 10 also had a genetic test for TMAU. One individual had a genetic test, but no urine test. Of those tested, 17 (52%) were diagnosed for TMAU, 5 (15%) with primary, 11 (33%) with secondary TMAU and 1 with unclassified TMAU (Table 1). Sixteen tested negative for TMAU. Twenty-three respondents reported experiencing problems or delays in being offered a diagnostic test for TMAU. The main problems encountered were a lack of awareness of the disorder by GPs (17 respondents) and reluctance to accept or recognise symptoms of body or breath malodour (8 respondents). Examples of free-text comments are given in Table 2.

**Table 2:** Problems in obtaining a diagnostic test

‘Difficult to convince medical practitioners of my symptoms especially as most were not aware of the disease Trimethylaminuria. It took me having an emotional breakdown and severe anxiety before I was reluctantly sent for a urine test.’
‘Finding a GP that believes you have the symptoms. Unfortunately, it seems likely to put you in a mental health hospital.’
‘tmau is so rare you cannot possibly have it’.
‘I experienced years of futile GP visits, being told that I don’t smell, to consider good hygiene practises, that I’m suffering from anxiety or imagining it.’
‘The dr told me what I was talking about didn’t exist.’
‘Doctors telling you basically implying that your crazy and need to see a psychiatrist.’
‘I was tested for STD. Given advice on personal hygiene, told it was all in the mind, was told they could not smell my odour, then put the fan on, opened windows and then covered nose.’
‘Symptoms not taken seriously and then multiple unnecessary diagnostic tests due to lack of awareness of the disease.’
‘When I attend my gp surgery the first time the doctor told me I was being paranoid.’
‘My GP was unaware about tmau and didn’t know what to do with me.’
‘GP said as there is no cure or treatment they can’t help me.’

### Problems associated with living with a body-odour condition

Of 40 respondents, all reported instances of bullying, harassment or ostracism in either educational settings, the workplace, while travelling or in their social life (Table 3). The highest incidences were in the workplace (36 respondents, 90%) and in social settings (35 respondents, 88%). Examples of negative experiences are given in Table 4. Recurring themes include accusations of poor hygiene, offensive comments and being shunned by work colleagues and the public.

**Table 3:** Experiences of bullying, harassment or ostracism

Setting	Number of respondents	Percent of respondents (%)
School	15	38
University	7	18
Other HE	12	30
Training course	14	35
Work place	36	90
Travel	26	65
Social life	35	88
Other*	10	25

*‘Other’ setting examples: accommodation, family, street, shopping, NHS, restaurant, cinema

**Table 4:** Bullying, harassment, ostracism and negative experiences

‘I have received insults, bullying, they have kicked me out of shops, neighbours have thrown stones to my windows and broke them or shout to me in street.’
‘Awful comments, people leaving immediately where I enter, complaining about me, spraying air freshener behind me.’
‘ostracism at work, name calling, people moving to another table in café.’
‘At work they spray air freshener when I pass. My family avoids inviting me to family gatherings and they talk behind my back that I smell very bad.’
‘Ostracised by work colleagues. Friends stopped inviting me out so I’ve lost many social contacts. Upsetting loud comments made on public transport and in shops by strangers.’
‘Trains, buses, people commenting on the smell and moving upstairs on the bus or away from me on trains. People crossing the street to avoid me.’
‘Regularly stressed at work in anticipation of an episode. I have drastically reduced by social life. Am highly anxious when I do go out with friends.’
‘Friends no longer want to hang out, it’s just awful’
‘Being treated as ‘unclean’, losing jobs’
‘Bullying at college. An episode in a restaurant which has cause me now to suffer with PTSD.’
‘Bullied from a young age for having Bad breath, at school no one wanted to be my friend. At work no-one wanting to sit next to me at lunchtime. Laughing behind my back, unable to express myself and unable to progress in my job, I have left work now.’
‘People spraying air freshener in the office around me whilst I’m sitting in my chair. People deliberately not sitting next to me. People blurting out random remarks relating to odour and smell in meetings. People coughing, closing their nose, giving angry looks. Being referred to as stinky.’
‘People in public transports next to me saying I smell like fish and laughing or making faces. To travel is really stressful for me. To be in a queue at the supermarket is stressfull. The enviroment at work would cause me not to be able to focus, go to the toilet to cry and to have a constant stress. My own ‘friends’ at high school making fun of me about this. People on highschool saying straight to me I should shower. I went to three different highschools, same results on the three of them - jokes, disgust faces, people who would be rude for no apparent reason, etc. This cause me two years of absolute depression not wanting to meet anyone or come out of bed or my home. For this I gave up two years of my studies.’
‘It was often commented on at school. I was also bullied a lot. I was repeatedly called into the manager’s office at work and told to do something about the smell. I have been asked if I shower, if I know how to shower properly and even if I knew I had to use soap.’
‘I’ve had people whispering and talking about me at work. Some just blatantly say that the place is stinking or there’s a fishy smell. I’ve had people call me names. I’ve had to change jobs lots of times because I can’t cope with the comments. I’ve had strangers make comments about my smell. It takes over your whole life and you end up constantly watching for people’s facial expressions or looks that they give to each other, or the whispered comments.’
‘Systematic bullying by co-workers, disrespect and negative comments from colleagues. Offensive comments from friends relating to personal hygiene issues.’
‘It has affected me in so many areas of my life and the experiences have stopped me completing, pursuing or trying things that are essential to progress in life and to be a healthy human being ie education, employment, relationships, social interactions, mental health to name a few.’

Thirty-nine respondents thought that their body/breath malodour symptoms had disadvantaged them in their daily life (Table 5). Again, the main categories in which disadvantage was perceived was in the workplace and in social settings (35 respondents, 90%, for both options). Examples of instances of disadvantage are given in Table 6. These include difficulties in being offered or retaining jobs and in forming and maintaining friendships and relationships, causing stress, fear and paranoia in work and social environments.

**Table 5:** Have your symptoms disadvantaged your daily life?

Setting	Number of respondents	Percent of respondents (%)
School	11	28
University	8	21
Other HE	11	28
Training Course	16	41
Work Place	35	90
Travel	23	59
Social Life	35	90
Other*	6	15

* ‘Other’ examples: accommodation, family, husband, marriage, friends, enjoying activities, medical

**Table 6:** Have symptoms disadvantaged your daily life?

‘I have been kicked out of places, ignored in queues, rejected in interviews.’
‘During my A levels it was a very tough time for me socially, I was very anxious about TMAU which was my main focus and not my studies. I did not feel comfortable talking to pastoral support about this due to embarrassment, fear of lack of understanding and further ostracism. Through school I struggled to maintain friendships and never really fit into a group.’
‘Particularly at work, unable to attend meetings. I no longer have a circle of friends and no socialising beyond my immediate family.’
‘Broke up Uni, struggle to find work, cannot work anymore with customer face to face, cannot live anymore with someone, no partnership/relationship, struggle to go shopping.’
‘I had to leave my job of 25 years due to physical and emotional bullying I gave up a good pension and have worked as a gardener ever since. My tmau symptoms have caused social anxiety, and I have missed out on a lot of social events.’
‘I’ve avoided joining clubs and other social groups for fear of embarrassment. I’ve lowered my expectations of friendships, relationships etc.’
‘My last time at work was stressful (a) because of the work itself and (b) because of the odour. I felt suicidal. I retired earlier than I would have done if not for this condition.’
‘It puts you off trying to achieve anything as you dread comments coming your way.’
‘Conscious and subconscious fear at work and in social life.
‘I used to go out to pubs and clubs and when the symptoms became very strong I tried everything and eventually had to stop going out completely, I had to change jobs constantly and try to get shorter working hours.’
‘I have lost work because of my odours. Also I have lost relationships because of the shock that I smell badly. I have not had a relationship for 20 years as I am afraid of the inevitable comments.’
‘Not going to family events, missing out Not shopping due to fear of being in queue.’
‘Social interaction become a problem as my confidence is non existent.’
‘I don’t want to attend any social functions, no one wants to talk to me either’.
‘Difficult to work and socialise with people when experience negative reactions and comments.’
‘I have avoided getting romantically involved with anyone.’
‘People do not treat me as a human being. The neighbours all discuss my smell. People move away from me all the time. I have no confidence in my life to go outside, even going outside to buy groceries is a difficulty. No one talks or sits next to me at work. I just try to hide as much as possible. I am constantly paranoid, I hate my life.’
‘Because people can smell my symptoms and make comments it has lead me to have social anxiety and now I have no friends and only go out to go to work.’
‘Some people wouldn’t make plans with me, other simply don’t speak to me.’
‘I moved to working nights which has further impacted opportunities available to me.’
I have isolated myself because I’ve become so paranoid on how people will react to me. I don’t smile as much anymore, I’m constantly crying, I feel suicidal at times, it comes to a point where you feel like what’s the point of going ahead in your life.’
‘I’ve had to leave some jobs that I’ve loved just because I couldn’t cope with the comments, whispers and looks from others. I am too terrified and paranoid to put myself back into that situation again.’
‘I’ve been dismissed from job after job.’
‘I tend to put myself in less social situations and isolate myself.’
‘fear of bullying determines where i place myself physically in relation to others at all times so i never choose to be in a room with others, unless i have no option. I long for a job i can do at home to avoid ‘public’ work and transport. I have stayed in very poorly paid jobs if they gave me less social contact. i didnt take driving lessons because of enclosed space in car. i never socialise now. shopping only online. cut own hair.’
‘Not enough attention during education settings due to my perceived smell. Additionally, also avoid social situations and people generally avoid me.’

Participants were then asked whether they thought that revealing their condition to employers, work colleagues or friends would be an advantage or a disadvantage. Many were not sure and there was no clear consensus (Table 7). Some respondents thought that it could be both an advantage and a disadvantage, depending on circumstances.

**Table 7:** Would revealing your condition be an advantage or disadvantage?

	Choice	Number of respondents
**Advantage**	Yes	13
	No	9
	Not sure	16
**Disadvantage**	Yes	11
	No	9
	Not sure	18

Table 8 gives examples of changes that participants thought would improve their life. Common themes include greater awareness and understanding of the condition by medical professionals, employers, fellow workers and the general public; better treatment and mental health support; acknowledgement that the odour is due to a medical condition and not a result of poor hygiene; and recognition of TMAU and other body odour conditions as an invisible disability.

**Table 8:** Changes that would improve quality of life

‘Understanding of my condition by doctors, by teachers and parents to educate children with sensibility about these kind of problems. Creating work laws that protect us versus unfair and illegal bullying at work. Tests more accessible and cheap. Creating more laws to recognize our illness as a disability.’
‘I think that classifying TMAU as a disability, and increasing its awareness to primary care doctors, health professionals and pastoral staff would help massively. It would be very helpful if I could leave work/school if I am having a flare up and make up the time at a later date. I think mental health support / counselling should be mandatory for people with TMAU.’
‘Having a medical report to be able to share with work or mention to friends so that people can believe I do have a genuine medical condition and not a hygiene issue.’
‘Yes, more understanding of the condition and how it affects patients, not just physically but the mental impact it has.’
‘A cure or a little hope that makes me have the life of a human being because this is like being buried alive.’
‘I would just like understanding and acceptance that I’m doing what I can to control a very difficult and poorly understood medical condition.’
‘If it was made a recognised disability and anyone who negatively commented were pulled up on it. If it was the same as racism or making comments at a person in a wheelchair.’
‘I am not sure if colleagues knowing about my condition would make things better. I have terrible shame about my symptoms.’
‘What I really want is to be able to work from home and shorter working hours at work.’
‘Support in the right food to eat. To have a group you can share with that understands the pain. For people to understand how this destroys you.’
‘Overall my life in general would become normal if able to manage or control this condition.’
‘A cure, better understanding by others, TMAU should be classed as a disability as it affects you in all aspects of your life, although it’s not visual.’
‘I feel if this was recognised as an invisible disease, more people would be more understanding and perhaps empathetic towards me and know that just because I smell bad isn’t because I don’t wash myself or I’m dirty but it’s because I have a disorder which causes me to smell bad.’
‘Nothing, only a cure for it could make this living hell any better. I just want to live a normal life.’
‘Being able to work from home, and so not have to interact with others.’
‘Possibly more comprehensive information on NHS websites and other health related websites could lead to a more widespread awareness of Trimethyalminuria and could facilitate understanding the causes and treatment - which might result in employers or colleagues developing a more sympathetic approach. If this were an option for everyone else diagnosed with Trimethylaminuria, as a recognised disability, then this could make life easier for other sufferers.’
‘But definitely there needs to be an understanding of the condition by medical people, employers, government, education system, welfare/benefits system and the general population.’

TMAU was originally called fish-odour syndrome and this term continues to be used by some individuals and organisations. Twenty-seven of 38 respondents (71%) considered this term to be derogatory and would like to see TMAU used in place of fish-odour syndrome, whereas 8 (21%) had no opinion. Many respondents thought the term ‘fish-odour syndrome’ to be embarrassing, humiliating, derogatory or disrespectful, with many commenting that the odour is not always fishy, but in some cases resembles a faecal or garbage odour.

The final question gave participants the opportunity to comment on any other issues regarding living with TMAU that they had not mentioned in other responses. Examples of such comments are given in Table 9. Common themes include loss of confidence, stress, exclusion, isolation, loneliness, depression and suicidal thoughts.

**Table 9:** Problems experienced with living with TMAU

‘Growing up I felt like I had no medical or pastoral support for my condition. I often felt guilty and as if it was my fault that I smelt badly, and often blamed myself for eating the wrong thing etc. Because TMAU is so rare and so unique I did not feel like I could compare myself to another group, or another disease which made me feel even lonelier. There was very little post-diagnosis support from the NHS. I think that TMAU has a huge impact on quality of life, and should be given the same attention as other conditions which also affect quality of life. There are serious psychological and social consequences of living with this condition and I think it does often prevent me living a fulfilled and productive life.’
‘The mental effect of the condition is far impacting than people may realise. In my case, I have considered suicide, more so at the onset as I couldn’t find any resources to assist. Having a contact with medical professionals who may be better placed to advise on the effect of some treatments or diets that are often tried in the hope of treating or minimising the odour, will be of a great help to all suffers. Many are desperately looking for help but can’t find any.’
‘More psychologists to help us have a more bearable mental health.’
‘The sense of isolation and fear of going out is hard to cope with sometimes. I would like to see more opportunities for support groups to aid speaking to and possibly meeting other sufferers. At times I’ve felt suicidal so this is probably the case for many others. It would be a real life line.’
‘This is a SOCIAL condition. We are not ill physically. Mentally, yes. I feel depressed and suicidal all the time. I feel like a leper and try to keep away from people. People look at me with disgust.’
‘I feel desperate and drained of energy in my search for help and support from medical professionals.’
‘It’s a hidden disability even though we look fit and healthy it impacts your life massively often causing you restrictions in work, family, travelling and being sociable.’
‘Mental health is so important, it took me a long time to get to a good state where I am able to function. This condition paralyzes all aspects of your life.’
‘My entire life has been absorbed by this condition and it is a daily struggle to live life fully and normally. Every area of my life is impacted, I am grateful to have this survey to express my feelings on how this condition affects us not only physically but mentally. I cannot express enough how important a cure is to me and my quality of life.’
‘I think more support is needed, especially with children, life is hard for them on a normal day, but to have this condition on top is so hard, and more help psychologically is desperately needed, with school counselling.’
‘I believe this is a such a horrible deliberating condition, it stops you from functioning as a normal person, you can’t eat, you can’t speak, you can’t work, you can’t socialise, you can’t do anything, it’s depressing, I am always paranoid and I’m exhausted.’
‘The hugely restrictive impossible diet to limit symptoms and lack of treatment are exceptionally difficult to live with.’
‘It really does affect every aspect of your life.’
‘I say a prayer every day we can find a cure so I can try and start living a normal life.’
‘I would like TMAU to be recognised and shared so that others can be made aware of it and understand more. Because so many people with tmau are being treated so badly and unfairly and suffering alone.’
‘This condition has totally ruined my life! I have no confidence, am paranoid, have depression and mood swings. I am terrified of social situations and of going back to work. I am tired of having to live this way and wish that there was finally a cure so that I can live the rest of my life like a normal person. A normal person who doesn’t have to worry about being the smelly one that everyone talks about!!’
‘If this condition became officially recognised for the terrible damage it causes to sufferers, that would help. That said,TMAU has destroyed my life, and the only hope remaining is that a cure is found.’
‘Everyone has pressures of life from time to time but TMAU makes living so much harder. It makes surviving so much harder. Every part of life is affected and the pressure is continuous.’

### Experiences of children living with TMAU

Parents or guardians of children with symptoms of TMAU were asked about problems encountered by the children. Three male children were reported to present with symptoms at 5 months, 1 or 12 years of age. Two had urine and genetic tests with a confirmed diagnosis of primary TMAU. The third child awaits tests after delays of almost two years. All three children had experienced bullying, harassment or ostracism in educational or social settings. However, one parent stated that once school staff understood the condition, they were very supportive. Points of concern raised by the parents/guardians are given in Table 10.

**Table 10:** Parent/guardian concerns

‘As a parent of a very young child with TMAU, I am very much able to shield him and protect him the best I can. The worry and anxiety I deal with daily about his future is very hard. I live in hope that there will be some kind of medication to regulated the smell on the near future. It is utterly heart-breaking as a parent to for your child to have this condition.’
**‘**Need more awareness of psychosocial impact of the condition General awareness of condition could be improved. More positive management rather than focussing on the negatives.’
‘Kids with this condition need more help and support. ‘

## Discussion

Although some survey participants reported good experiences of their interactions with health professionals, the majority experienced problems or delays in being offered a diagnostic test for TMAU. One reason for this could be that, because of the episodic nature of the malodour [7], symptoms may not present during a medical consultation and, thus, the individual is not believed, leading to the assumption that the malodour is imaginary. In addition, some individuals are unable to detect the smell of TMA [22]. Another contributing factor is that the urine test for TMAU, i.e., the measurement of TMA and TMAO, is complicated by the volatile nature of TMA. The measurement involves the use of sophisticated equipment and requires skilled, experienced personnel, and is not routinely available on the NHS.

Currently, the NHS has no standardised pathway for the diagnosis of TMAU. When a patient presents to a GP with a body malodour syndrome, there is no guidance on how to investigate, diagnose or manage TMAU. Without a pathway, the referral process to a specialist is opaque and often inaccessible. Furthermore, the genetic test for mutations in the *FMO3* gene is not available on the National genomic test directory for rare and inherited diseases [23], unlike genetic tests for other inborn errors of metabolism. Unfortunately, genetic counselling for individuals with primary TMAU is not routinely offered.

It would be an advantage to establish a standardised pathway from primary care to a specialist unit with access to a robust and reliable test and diagnostic criteria, with genetic testing if required. Establishing a robust and reliable diagnostic test would allow clinicians to distinguish between malodour conditions caused by TMAU and those of other origin. Indeed, almost half of those tested (48%) were negative for TMAU. Although some of the negative results could be due to problems with the test, it is likely that in some of these cases the malodour is caused by conditions other than TMAU and should warrant further investigation. It has been reported that some of those with body malodour symptoms do not suffer from TMAU [24] and the focus on TMAU has diverted attention away from the understanding of other body odour disorders. Patients want a diagnosis of their condition and clinicians should help facilitate this.

Irrespective of the diagnosis, all of the malodour sufferers reported that they had been bullied, harassed or ostracised in educational, workplace or social settings. As a consequence, all respondents considered that they had been discriminated against and hence disadvantaged, severely curtailing their life chances. Parents/guardians indicated that all of the affected children had faced instances of bullying and/or ostracism in educational or social settings.

The earliest report of an individual with symptoms reminiscent of TMAU comes from the great Indian epic the *Mahabharata*, compiled in about 400 CE [25]. The affected individual, the young woman Satyavati was cast out from society and condemned to the solitary life of a ferry woman. To this day, social isolation remains the fate of those affected by body and breath malodour conditions.

Our study reveals that those affected by malodour conditions experience considerable stress, anxiety and fear of being in close proximity to others, leading to feelings of loneliness, depression and, in extreme cases, suicidal thoughts. Respondents would welcome greater mental health support to help them cope with these issues. Understanding by primary care physicians and mental health teams of the underlying aetiology of the mental health problems could lead to more effective management.

Respondents thought that their lives could be improved by a greater awareness of malodour conditions by medical professionals, employers, work colleagues, family members and the general public. Also considered important was recognition that the malodour was not their fault, but due to a medical condition.

There are few treatment options for malodour conditions. For TMAU, these include dietary component restrictions [7, 26, 27], which can be severe and difficult to maintain. Consequently, there is a need for improved treatment and management plans for affected individuals [28]. Primary TMAU is caused by mutations that affect the oxygenating function of FMO3. Because FMO3 is involved in the metabolism of a variety of therapeutic drugs [13, 29], care should be exercised in prescribing drug substrates of FMO3 to those affected by primary TMAU, to minimise the likelihood of an adverse drug response. For both primary and secondary TMAU, symptoms could be worsened by the competition of the drug substrate with TMA for residual functional FMO3.

One of the outcomes of the survey is the wish of participants that TMAU be classified as a disability, offering affected individuals the same rights under the Equality Act 2010 as those afforded to people with recognised disabilities, thus protecting them against discrimination. This would require representation to government for a policy change.

## Conclusions

The survey findings reveal that people do not generally sympathise with someone with body and breath malodour. Living with the disorder can cause immense hardship and distress, both mentally and socially. TMAU and body odour symptoms have devastating effects on the quality of life of individuals in terms of their ability to interact with society in their personal, educational and working lives, demonstrating that societal effects of a metabolic disorder can lead to isolation and loneliness. There is a need to recognize malodour disorders as invisible disabilities and to classify primary TMAU as an inborn error of metabolism with the same access to referrals and diagnostics as other inherited disorders.

## Data Availability

Data are presented in the paper.

## Abbreviations

CE: Common Era
FMO3: Flavin-containing monooxygenase 3
MEBO: Metabolic Body Odours
TMA: Trimethylamine
TMAO: Trimethylamine *N*-oxide
TMAU: Trimethylaminuria
